# Arsenic Species and Nitrogen Stable Isotope Ratios in the Japanese Diet—Dietary Markers of Seafood

**DOI:** 10.3390/foods15030500

**Published:** 2026-02-01

**Authors:** Jun Yoshinaga, Tomohiro Narukawa

**Affiliations:** 1 Faculty of Life Sciences, Toyo University, 48-1 Oka, Asaka 351-8510, Saitama, Japan; 2 National Metrology Institute of Japan, National Institute of Advanced Industrial Sciences and Technology, 1-1-1 Umezono, Tsukuba 305-8563, Ibaraki, Japan

**Keywords:** nitrogen stable isotope ratio, arsenobetaine, duplicate diet

## Abstract

Interest in seafood diet and health warrants a biomarker for seafood consumption. Nitrogen isotope ratio (^15^N/^14^N, expressed as δ^15^N (‰)) has been regarded as a biomarker for such a purpose. This study aims to elucidate the applicability of levels of arsenobetaine (AB), a non-toxic organic arsenic compound, in the diet as a marker of seafood abundance because of its known distribution in marine animals. The concentrations of AB and other arsenic species and δ^15^N in duplicate diet samples collected from 150 Japanese adults were analyzed for a possible relationship with the inclusion of seafood and seaweed in the diet samples. Information was collected from the menu reported from the duplicate diet donners, and a possible correlation between the levels of AB and δ^15^N was tested. As expected, median levels of AB and δ^15^N were more elevated in the duplicate diet that contained seafood (54.6 ng/g dry and 3.60‰) than that without seafood (<7 ng/g dry and 3.01‰). Additionally, there was a significant positive correlation between the two components (Spearman’s *ρ* = 0.384, *p* < 0.001). The distinct difference between the seafood-containing and non-containing diet suggested that the AB content of the diet is a more sensitive marker of seafood abundance than δ^15^N.

## 1. Introduction

Arsenic (As) is found at higher concentrations in marine organisms, including plants and animals. However, the species of water-soluble As in marine organisms are different between marine plants and animals—animals predominantly contain arsenobetaine (AB) [1,2,3,4] while plants do not contain AB but different As-containing compounds like arsenosugar [5,6,7,8], both of which are virtually non-toxic. This general tendency holds true to water-soluble As species in marine organisms for human consumption [9]. Thus, the occurrence of As species in the diet of a population can vary both quantitatively and qualitatively depending on the food habits of the population. For instance, the diet of people who do not consume marine products would generally contain low levels of As with limited As species, e.g., inorganic As (iAs) and some methylated compounds like dimethylarsinic acid (DMA) [10,11], from foods of terrestrial plant and animal origins. The diet of people who consume marine products, such as the Japanese, contains AB and other seaweed-related As compounds in addition to iAs and DMA, of which the composition can vary depending on the relative abundance of seafood and seaweeds in the diet. It is expected that a seafood-abundant diet would contain AB at a higher level.

Nitrogen stable isotope ratio (^15^N/^14^N) has been used as a marker for the trophic level of organisms in previous ecological studies [12,13], where animals preferentially excrete a lighter isotope and thus the heavier isotope accumulates in the body [14]; therefore, the isotope ratio, conventionally expressed as δ^15^N, becomes more elevated along the food chain [15]. Since fish and other marine animals, such as crustaceans and carnivorous mollusks, are generally at a higher trophic level in the aquatic environment, seafood has elevated δ^15^N compared to terrestrial animals [16]. Therefore, a diet abundant in seafood would have elevated δ^15^N; however, it has not been confirmed to date. Based on this presumption, δ^15^N has been extensively applied to the estimation of marine resources dependency of ancient populations and individuals by using excavated bone collagen [17,18,19]. Studies on the intake of seafood with δ^15^N of biological samples have also been highlighted in contemporary populations in relation to diet and diseases [20,21,22].

Taking into consideration the As species and δ^15^N of seafood for human consumption, it is expected that levels of AB concentration as well as δ^15^N would be higher in a diet with abundant seafood, and that the two components would positively correlate with each other in the diet. It is the aim of this study to confirm this relationship in the Japanese diet, which has been assumed but not confirmed in previous studies. Since more and more studies focus on the association between health and seafood intake [20,21,22], the demand for a suitable and diverse biomarker of seafood intake is increasing. As a preliminary stage of the establishment of the biomarker of seafood intake, the establishment of a dietary marker of seafood is warranted. This study was carried out to examine if AB is a suitable marker of seafood in the diet.

## 2. Materials and Methods

### 2.1. Overview of the Study

**Figure 1:**
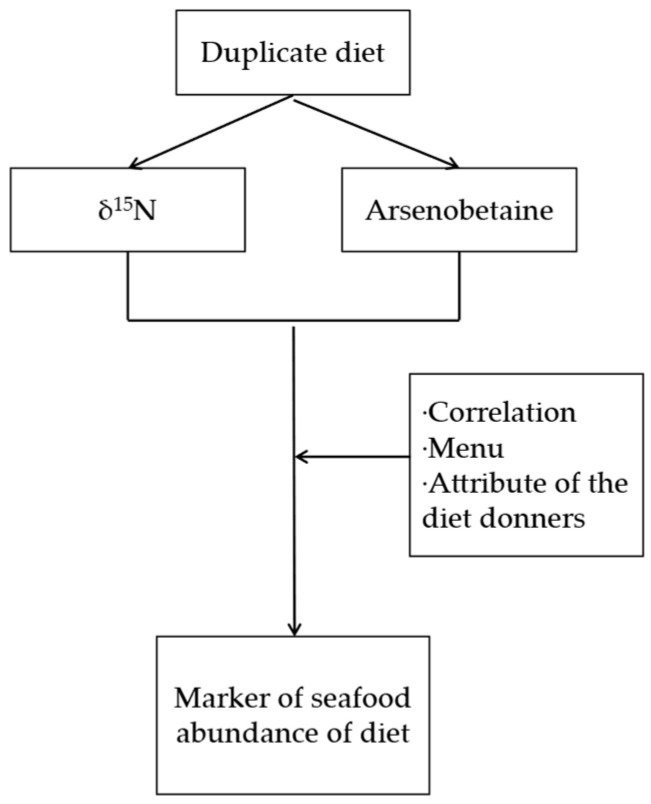
Overview of this study.

Figure 1 presents the overview of this work. This work statistically analyzed the association between δ^15^N and AB content of duplicate diet samples collected from 150 Japanese adults, both of which have already been published in previous works [23,24,25]. The association, together with some information on diet, is analyzed to see if AB content is a marker of seafood abundance in the diet, as is δ^15^N. The duplicate diet samples and analytical methods are described briefly in the following sections.

### 2.2. Duplicate Diet Samples

The duplicate diet samples were collected from participants of the previous study on the potential association between intake and urinary excretion of iAs and methylated As in the Japanese [23,24]. Briefly, a 24 h duplicate diet was collected from 150 Japanese adults (65 males and 85 females, mean age: 44.9 years old) during 2017–2018. The participants were asked to prepare two portions of all of the foods and beverages on sampling day, and they chose and to put one portion into polypropylene bottles of known weight. The samples were sent to Toyo University under refrigeration. The participants were also asked to provide a menu of their duplicate diet sample. The duplicate diet sample was then homogenized in one lot in a large-volume food processor. A portion of the homogenate was freeze-dried and pulverized for the following chemical analysis.

The study was approved by the Ethical Committee of Toyo University (TU2017-005 and TU2023-016).

### 2.3. Analysis

Arsenic speciation analysis of the 150 samples was carried out by high performance chromatography–ICP mass spectrometry after the extraction of the freeze-dried duplicate diet samples with 0.07 mol/L hydrochloric acid containing 0.01% pepsin (synthetic gastric juice). Concentrations of iAs, methylarsonic acid (MMA), dimethylarsinic acid (DMA), and arsenobetaine were determined [23,24] (see Supplementary Materials). The δ^15^N of the sample was determined by isotope ratio mass spectrometry after defatting the freeze-dried sample with chloroform + methanol [25] (see Supplementary Materials). The δ^15^N was calculated by the following conventional equation:

δN15‰= N15/N14sample−N15/N14standardN15/N14standard×1000

The standard for *δ*^15^*N* is atmospheric N_2_.

All of the analyses were carried out under rigorous internal and external quality control practices by using certified reference materials (CRMs) of food and diet matrices, which were described in detail in each of the articles [23,24,25] and in the Supplementary Materials. Note that As species were extracted from the samples with synthetic gastric juice in this study, but the species were quantitatively extracted, which was proved by the consistent results with the certified values of As species concentrations in the CRMs used [23,24].

### 2.4. Statistical Analysis

The concentrations of As species and δ^15^N in the duplicate diet were compared by age, age groups (4 groups, 20-year intervals), and gender of the participants with the U-test or Kruskal–Wallis test. Correlation between the As species concentrations and δ^15^N was examined by Spearman correlation analysis. The As species concentrations and δ^15^N were compared between inclusion and no-inclusion of seafood or seaweed, for which information was taken from the menu of the duplicate diet the participant provided. All of the analyses were conducted using SPSS ver. 29.0 (IBM, Tokyo, Japan).

## 3. Results

The mean and median concentrations of iAs, MMA, DMA, and AB with standard deviation and min–max range in the diet samples are presented in Table 1. In this table, dry weight basis concentrations of As species are shown because the aim of this study was to correlate with δ^15^N. Detection limits for MMA, DMA, and AB were 6, 6, and 7 ng/g, respectively, and detectable diet samples for MMA, DMA, and AB was 0%, 64%, and 74%, respectively. Inorganic As was detectable in all of the samples. Mean and standard deviation for DMA and AB were calculated by substituting the concentration in undetectable samples with 1/2 of the detection limit values and are shown in this Table. MMA was excluded from the following statistical analysis. Descriptive statistics for δ^15^N are also shown in this table, which was recapitulated from our previous study [25]. The concentrations of As species and δ^15^N were not normally distributed (Kormogorov–Smirnov test, *p* < 0.05); the results of the non-parametric statistical method are presented hereafter.

**Figure 2:**
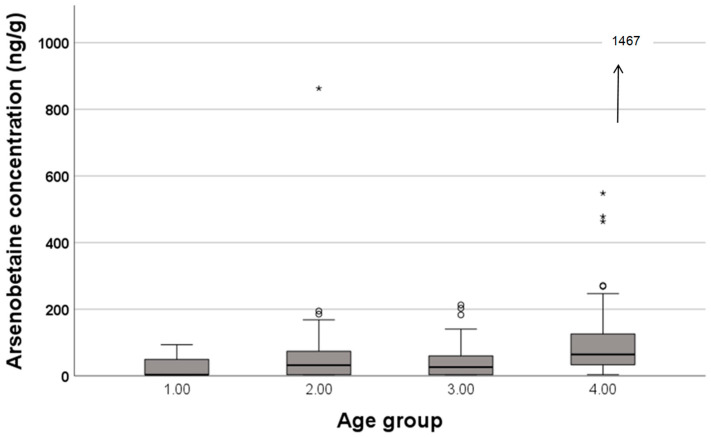
Variation in arsenobetaine concentration in the duplicate diet according to the age group of the duplicate diet donors. Age group 1: <20; 2, 21–40; 3, 41–60; 4, >61 yrs. Open circle and asterisks represent outlier values, i.e., greater than 1.5 and 3 times the interquartile range, respectively. Arrow indicates the value is plotted out of the vertical axis of this figure.

No gender difference was found for the concentrations of As species and δ^15^N. A significant correlation with age was found only for AB concentration (*ρ* = 0.265, *p* < 0.001). Variation with age group (0–20, 21–40, 41–60, >61) was significant for AB concentration (*p* < 0.001, Kruskal–Wallis test) and iAs concentration (*p* = 0.002, Kruskal–Wallis test). The concentration of iAs varied non-linearly with age groups, while AB concentrations showed an increasing tendency according to age group (Figure 2).

**Figure 3:**
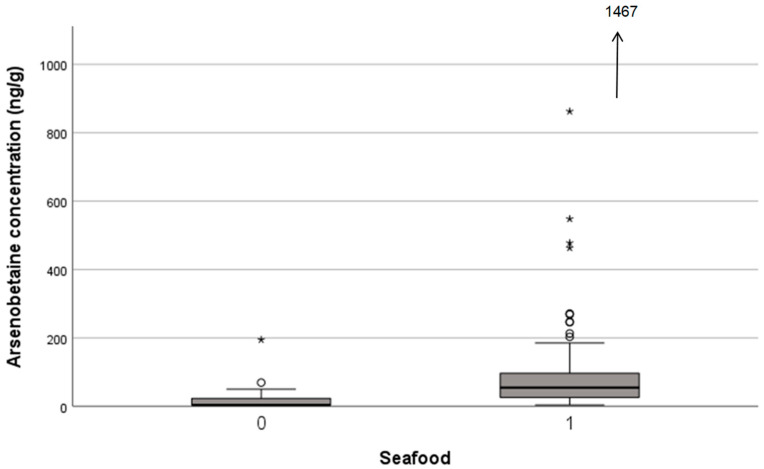
Comparison of arsenobetaine concentration in the duplicate diet between the seafood-containing diet and the seafood-free diet. The difference was statistically significant (*p* < 0.001, U-test). Marks in the figure are the same as those described in the footnote to Figure 2.

The concentration of AB and δ^15^N was significantly higher in the duplicate diet that contained seafood (*n* = 111) than in the one that did not contain seafood (*n* = 39) (Table 2). Median concentrations of AB in the seafood-containing diet and that in the non-seafood-containing diet were 54.6 ng/g and <7 ng/g, respectively (Figure 3). The δ^15^N of seafood-containing and non-containing diets was 3.60 and 3.01‰, respectively, as reported in the previous study [25]. The concentrations of iAs and DMA did not differ between the containing/not-containing seafood diet. The concentrations of DMA and iAs were significantly higher in the duplicate diet that contained seaweed (*n* = 87) than in the one that did not contain seaweed (*n* = 63). Median concentration of DMA in the duplicate diet that contained seaweed and that did not contain seaweed were 17.2 ng/g and 9.1 ng/g, respectively. Median concentrations of iAs in the duplicate diet that contained seaweed and that did not contain seaweed were 66.9 ng/g and 48.3 ng/g, respectively. The concentrations of AB and δ^15^N did not differ by the inclusion/non-inclusion of seaweed.

**Figure 4:**
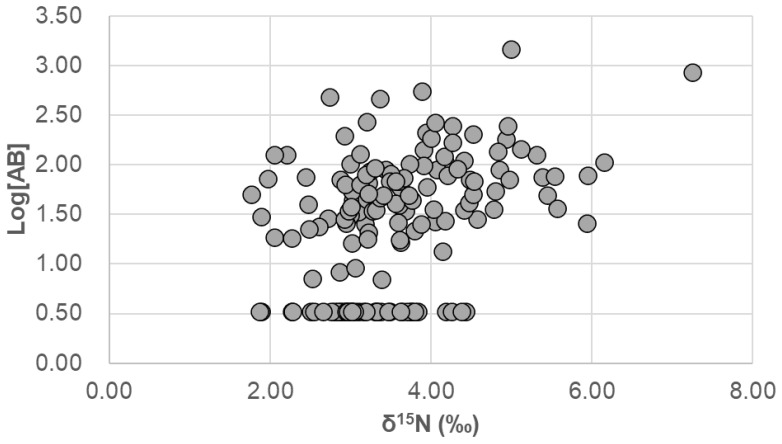
Scatter plot of dietary δ^15^N-arsenobetaine concentration. The correlation was significant (*ρ* = 0.384, *p* < 0.001, Spearman correlation analysis).

Correlation between As species concentrations and δ^15^N was analyzed by using Spearman analysis. The positive correlation between δ^15^N and AB concentration (*ρ* = 0.384, *p* < 0.001) and that between δ^15^N and DMA concentrations (*ρ* = 0.183, *p* < 0.05) were significant, but the correlation between δ^15^N and iAs was not significant (*ρ* = 0.032, *p* > 0.05). Since there was a significant positive correlation between the concentrations of DMA and AB (*ρ* = 0.283, *p* < 0.001), partial correlation analysis using log-transformed concentrations was employed to find that the correlation between AB and DMA resulted in a seemingly significant DMA-δ^15^N correlation. Figure 4 presents scatter plots of δ^15^N-AB concentration. Since the variation in AB concentrations was large, AB concentration is expressed as a log-transformed value in Figure 4.

## 4. Discussion

The levels of As species and δ^15^N in duplicate diet samples were consistent with our prediction to a large extent—elevated AB and δ^15^N levels in duplicate diets that contained seafood compared to those that did not contain seafood, and higher DMA and iAs concentrations in the duplicate diet that contained seaweed than those that did not contain seaweed (Table 2).

Although the significant difference in δ^15^N of the present duplicate diet samples with/without seafood was already reported in our previous study [25], the result for AB is a new finding. In the previously published studies, AB was found in marine animals, including fish, shellfish, and crustaceans, at high concentrations (mostly 1 μg As/g wet weight, up to 30 μg As/g wet weight). However, it was found in freshwater fish at lower concentrations [9,26]. Meanwhile, it was undetectable in terrestrial organisms; some species of fungi were found to be an exception [27,28]. The content of AB in edible mushrooms was reported to be up to 0.9 μg As/g wet weight [27]. However, taking the generally negligible abundance of mushrooms in the whole diet, seafood is virtually the only significant contributor of AB to the human diet. In this respect, our present finding could be predicted from previous knowledge, and AB may be a candidate as a marker of seafood abundance in the diet; however, to date, it has not been used in this capacity, as far as we are aware. Recently, Stråvik et al. [29] for the first time suggested urinary AB levels as a suitable biomarker of seafood consumption in pregnant women. The present result supports Stråvik et al.’s findings from a diet point of view.

It has been found that the δ^15^N of fish is the most elevated among other food categories [16,30]; therefore, the significantly elevated δ^15^N of a diet containing seafood than that not containing seafood is reasonable, as we have discussed in the previous study [25]. As a result, and as expected, δ^15^N and AB concentration were significantly positively correlated (Figure 4) due to preferential excretion of ^14^N in animals [14] and accumulation of AB in the aquatic food web [1,2] that results in the elevated levels of δ^15^N and AB content in fish and carnivorous mollusks. This correlation indicates that both AB content and δ^15^N of diet are suitable markers of seafood abundance in the diet. Meanwhile, the correlation coefficient (*ρ* = 0.384) seemed to be smaller than we assumed: the coefficient indicates that only 15% of the variance of the one variable can be explained by the other. In addition, the difference in δ^15^N between the duplicate diets that contained seafood and that did not contain seafood (3.80 vs. 3.01‰) was rather small, while the difference in AB was more distinct (54.6 vs. <7 ng/g).

Yoshinaga [25] pointed out that δ^15^N of the duplicate diet from elder participants was not elevated, though the duplicate diet of elder participants contained seafood more frequently than the diet of younger participants (Table 3). The author ascribed this lack of significant elevation of δ^15^N in the duplicate diet from elder participants to the effects of other foods with higher δ^15^N, such as meat. The national statistics revealed that elderly Japanese people consume more seafood and less meat [31]. However, as shown in this study, AB concentrations in the duplicate diet increased towards higher age groups (Figure 2), and they were also significantly positively correlated with the age of the participants (*ρ* = 0.265, *p* < 0.001). Thus, AB concentration is suggested as a more sensitive marker of seafood abundance of diet than δ^15^N.

The somewhat inferior sensitivity of δ^15^N found in this study may partly be due to the defatting of the duplicate diet sample employed as a pretreatment of δ^15^N measurement: previous studies found that defatting increased δ^15^N of aquatic organisms, including fish [32,33], probably due to the removal of polar amino acids. If the effect of defat on δ^15^N was variable depending on the duplicate diet samples, then it could have obscured the ability of δ^15^N for the estimation of the seafood abundance of the diet.

The higher DMA and iAs concentrations in the diet with seaweed (Table 2) are ascribed to the fact that seaweed contains DMA at higher concentrations than other foods [34], and seaweed Hijiki is a seaweed that contains iAs at considerably high levels [35,36], and that is consumed by the Japanese daily.

It must be noted that the statistical analyses and their interference were based on qualitative but not quantitative information on seafood/seaweed abundance in the duplicate diet. It was based on menu information without data on the amount and species of seafood contained in the diet. This lack of quantitative information could have obscured the actual relationship. It is warranted to examine the association between quantitative seafood abundance and AB or δ^15^N of a diet in the future.

## 5. Conclusions

It was suggested that levels of AB, in addition to δ^15^N, can be a marker of seafood abundance in the Japanese diet; moreover, AB seems to be a more sensitive marker than δ^15^N. Since the biomarker of seafood consumption, e.g., levels in biological fluids or hair, would be increasingly used for the studies on diet and health in other parts of the world, the quantitative confirmation of the presumption that seafood abundance in diet is actually related to the level of AB, in addition to δ^15^N, is warranted in other parts of the world, as well as in Japan.

## Figures and Tables

**Table 1:** Concentrations of As species (dry wt. basis) and δ^15^N in duplicate diet samples (*n* = 150).

	iAs (ng/g)	MMA (ng/g)	DMA (ng/g)	AB (ng/g)	δ^15^N (‰)
Detection frequency (%)	100	0	64	74	-
Mean	83.6	<6	17.2	77.1	3.58
SD	94.0	-	14.3	156.7	0.93
Median	60.2	<6	14.8	35.9	3.42
Min–Max	2.9–652	-	<6–66.5	<7–1467	1.77–7.25

Abbreviations: SD, standard deviation; iAs, inorganic arsenic; MMA, methylarsonic acid; DMA, dimethylarsinic acid; AB, arsenobetaine.

**Table 2:** Median of As species concentrations and δ^15^N in duplicate diets that contain or did not contain seafood and seaweed.

	Seafood			Seaweed		
	Contain (*n* = 111)	Not Contain (*n* = 39)	U-Test	Contain (*n* = 87)	Not Contain (*n* = 63)	U-Test
iAs, ng/g	56.9	62.2	*NS*	66.9	48.3	*p* < 0.01
DMA, ng/g	15.0	12.2	*NS*	17.2	9.1	*p* < 0.05
AB, ng/g	54.6	<7	*p* < 0.001	41.0	27.3	*NS*
δ^15^N, ‰	3.60	3.01	*p* < 0.001	3.41	3.44	*NS*

Abbreviations: *NS*, not significant. Others are identical to those in the footnote of Table 1.

**Table 3:** Number of duplicate diet samples according to the age group of participants and containing/not-containing seafood.

		Age Group			
		0–20 yrs	21–40 yrs	41–60 yrs	<61 yrs
Seafood	Contain	5	35	29	42
	Not contain	6	21	8	4

χ^2^ = 16.036, *p* < 0.001.

## Data Availability

The data presented in this manuscript are available on request from the corresponding author.

## Supplementary Materials

The following supporting information can be downloaded at: https://www.mdpi.com/article/10.3390/foods15030500/s1, Table S1: Demographic summary of the subjects of the present study; Table S2: External quality assurance of inorganic As analysis employed in this study.
