# Supplementary material for: Arsenic Species and Nitrogen Stable Isotope Ratios in the Japanese Diet—Dietary Markers of Seafood
Source: Foods. 2026 Feb 1;15(3):500. doi: 10.3390/foods15030500 (PMC12896717; doi:10.3390/foods15030500)
Supplement: Supplementary file 1 [file foods-15-00500-s001.zip › foods-4015637-supplementary.pdf]

Supplementary Materials for Yoshinaga and Narukawa, Arsenic Species and Nitrogen Stable Isotope Ratios in Japanese Diet—Dietary Markers of Seafood

1. Duplicate Diet Sampling and Preparation (Yoshinaga and Narukawa, 2020) [1]

1.1. Subjects

The 150 subjects consisted of 65 males and 85 females of mean age 44.6 and 45.2 years old, respectively, who responded to personal recruitment and brochure distributed with local newspaper. Inclusion criteria was that those who are adult Japanese and who were willing to donate duplicate diet by the designated procedure. No exclusion criteria were set. Table S1 shows gender/age distribution of the present subjects. Most of the subjects resided in Kanto area (greater Tokyo Metropolitan area) but some did in other parts of Japan. The occupation of the subjects included student, house wife, office worker, and no-job (retired).

Table S1. Demographic summary of the subjects of the present study.

	**Male**	**Female**	**Difference**
**Number of subjects**			
Age <30 yrs	21	25	
Age 30–59	25	33	*ns*
Age 60<	19	27	
Total	65	85	
Mean age (SD)	44.6 (20.2)	45.2 (20.0)	*ns*
Mean body weight, kg (SD)	65.9 (8.3)	54.6 (8.4)	$p<0.05$

1.2. Duplicated Diet Urine Sampling

Four polyethylene containers (ZipLok, 1.1 L) for solid food samples and two 2-L polypropylene bottles for liquid food/beverage samples were distributed to the subject to collect duplicate diet. The subject was also asked to record the menu of duplicate diet sampling day. The containers and bottles were pre-weighed.

All the plastic bottles were acid-washed prior to use except for ZipLoc container which was used without prior washing. It was confirmed that iAs leaching from ZipLoc container was not detectable with inductively coupled plasma (ICP) mass spectrometry (0.15 mol/L HNO_3 extractable As: <0.3 ng/container).

1.3. Diet Sample Pretreatment

Upon receipt of duplicate diet sample from subject, the containers and bottles were weighed and total duplicate diet weight was calculated by subtracting the weight of empty containers and bottles that were pre-weighed. The content of the containers and bottles were mixed in one lot, and then homogenized in a food processor (Cuisinert, Tokyo, Japan). Since beverage was included in all of the DD samples, addition of extra water to facilitate homogenization was not necessary. Approximately 60 g of the homogenized sample was taken in a polypropylene bottle and freeze-dried for 3–7 days. After freeze-drying, weight loss was measured and recorded as moisture content of duplicate diet sample. The freeze-dried material was pulverized with an agate mortar and pestle, and the powder was stored at 4 ^{o}C for subsequent iAs analysis.

2. Analytical Method of As Species in Duplicate Diet (Yoshinaga and Narukawa, 2021) [2]

Arsenic species in duplicate diet sample were extracted from 1 g of freeze-dried sample in 20 g of 0.07 mol/L HCl containing 0.01% (w/v) pepsin, prepared from Ultrapure HCl (Kanto Chemical Co. Inc., Tokyo, Japan) and pepsin from porcine gastric mucosa powder (250 units/mg solid, Sigma-Aldrich Japan G.K., Tokyo, Japan) in polypropylene

tube. The sample in the synthetic gastric juice was shaken with a reciprocal shaker for 2 h at room temperature. After shaking, the tube was centrifuged (4000 rpm for 10 min), and the supernatant was filtered through 0.45 μm PVDF membrane filter. The filtered supernatant was diluted five times with Millipore water and injected to HPLC.

The concentrations of As(III), As(V), MMA, DMA, and AsB in the filtered extracts of duplicate diet sample were determined by HPLC-ICP mass spectrometry (HPLC-ICP-MS). An HPLC (Nanospace SI-2, Osaka Soda Co., Ltd., Osaka, Japan) system equipped with a CAPCELL PAK C 18 MG column (length 150 mm, internal diameter 4.6 mm, Osaka Soda) was used for the separation of the As species, and ICP-MS was Agilent 7500-ce (Agilent Technologies, Tokyo, Japan). Chromatographic conditions and ICP-MS operational conditions were described in detail elsewhere.

Standard solution for As(III) was purchased from Kanto Chemical Co. Inc. (Tokyo, Japan), of which traceability of As concentration was guaranteed by Japan calibration service system (JCSS). Standard solution for MMA was prepared from commercially available reagent (Tri Chemical, Yamanashi, Japan). The concentration of MMA stock standard solution was calibrated against the JCSS guaranteed As solution after its purity had been evaluated. As(V), DMA and AsB certified reference materials, namely NMIJ CRM 7912-a As(V) Solution, NMIJ CRM 7913-a Dimethylarsinic Acid Solution and NMIJ CRM 7901-a Arsenobetaine Solution (NMIJ/AIST, Ibaraki, Japan), were used as source standard solutions for As(V), DMA and AsB, respectively.

Note that concentrations of As(III) and As(V) were summed and used as iAs for duplicate diet sample analysis because transformation of valency could take place during extraction procedure. Detection limits for iAs, MMA, DMA, and AsB were 0.06–0.07 ng As/g in solution or 6.1–6.8 ng As/g dry weight in diet sample.

Results of the external quality assurance by suing certified reference materials are shown in Tables S2 and S3.

Table S2. External quality assurance of inorganic As analysis employed in this study.

	**NMIJ CRM 7405-a Hijiki Seaweed**		**NMIJ CRM 7502-a White Rice Flour**	
	Certified	**Measured (n = 8)**	**Certified**	**Measured (n = 8)**
As(V)	10.1 ± 0.5	10.1 ± 0.3		
As(III)+As(V)	—		0.098 ± 0.006	0.096 ± 0.004

3. Analytical Method of $\delta^{15}N$ of Duplicate Diet (Yoshinaga 2025) [3]

An aliquot (500 mg) of the freeze-dried sample was defatted in 5 mL of chloroform:methanol (2:1) by sonication and the solvent was discarded after centrifugation. This was repeated two more times or until the solvent became clear and then washed with acetone twice. All of the solvents used were of HPLC grade from Kanto Chemicals Co. Inc. (Tokyo, Japan). After evaporating the residual acetone at 55°C, the samples were sent to Shoko Science Co Ltd. (Saitama, Japan) for carbon and nitrogen stable isotope ratio measurement, where elemental analyzer (Flash EA1112, Thermo Fisher Scientific, Kanagawa, Japan)/isotope ratio mass spectrometer (Delta V Advantage, Thermo Fisher Scientific) was used for the measurement with amino acids (alanine, glycine, and histidine) as working standards. Three alanine with different isotope ratios were used for nitrogen isotope ratio determination, which had been calibrated against two IAEA standards (IAEA N-1 and N-2). The estimated uncertainty of the calibration with the alanine as laboratory standard was 0.14 ‰ when uncertainty of the certified values of IAEA standards (0.07 and 0.12 ‰ (k=1) for IAEA N-1 and N-2, respectively) and repeatability of nitrogen isotope ratio measurements of the alanine are taken into consideration. The stable isotope ratios of nitrogen wwas expressed as conventional $\delta^{15}N$ notation (‰).

A certified reference material (CRM) of duplicate diet matrix, NIES CRM No. 27 Typical Japanese Diet from the National Institute for Environmental Studies (Ibaraki, Japan), prepared for quality assurance of trace element analysis, was defatted as was done for duplicate diet samples and measured for $\delta^{15}N$ in each batch of isotope ratio measurement as an internal quality assurance practice. The result wwas5.60±0.08 ‰ for $\delta^{15}N$, respectively (n=11 each), indicating that the present isotope ratio analysis was satisfactorily precisely done.

External quality control was not possible because no certified reference material of diet matrix with certified $\delta^{15}N$ values is available.

Table S3. Anaytical results of organic arsenicals in food-related CRMs (mg/kg dry wt unless otherwise indicated).

	**Monomethylarsonic Acid (MMA)**		**Dimethylarsinic Acid (DMA)**		**Arsenobetaine (AsB)**	
	**Measured***	**Certified**	**Measured***	**Certified**	**Measured ***	**Certified**
NMIJ CRM 7402-a Cod					35.8 ± 0.5	35.5 ± 1.8
NMIJ CRM 7403-a Swordfish					6.23 ± 0.10	6.23 ± 0.21
NMIJ CRM 7532-a Brown Rice			0.0183 ± 0.0005	0.0186 ± 0.0008		
NMIJ CRM 7533-a Brown Rice			0.092 ± 0.002	0.092 ± 0.004		
NIST SRM 3035 Apple Juice **	9.39 ± 0.27	9.38 ± 0.64	5.23 ± 0.14	5.23 ± 0.26		

* Mean ± SD of n = 6 measurements. ** Unit: µg/kg.

REFERENCES

1. Yoshinaga, J.; Narukawa, T. Association of dietary intake and urinary excretion of inorganic arsenic in the Japanese subjects. *Regul. Toxicol. Pharmacol*. **2020**, *116*, 104745.
2. Yoshinaga, J.; Narukawa, T. Dietary intake and urinary excretion of methylated arsenicals of Japanese adults consuming marine foods and rice. *Food Addit. Contam*. **2021**, 38, 622–629.
3. Yoshinaga, J. Carbon and nitrogen isotopic composition of duplicate diet of the Japanese. *Rapid Commun. Mass Spectrom*. **2025**, e10014.
